# Differences in Oral Structure and Tissue Interactions during Mouse vs. Human Palatogenesis: Implications for the Translation of Findings from Mice

**DOI:** 10.3389/fphys.2017.00154

**Published:** 2017-03-15

**Authors:** Kai Yu, Mei Deng, Theresa Naluai-Cecchini, Ian A. Glass, Timothy C. Cox

**Affiliations:** ^1^Department of Pediatrics, Division of Craniofacial Medicine, University of WashingtonSeattle, WA, USA; ^2^Center for Developmental Biology and Regenerative Medicine, Seattle Children's Research InstituteSeattle, WA, USA; ^3^Birth Defects Research Laboratory, Department of Pediatrics, University of WashingtonSeattle, WA, USA; ^4^Department of Anatomy and Developmental Biology, Monash UniversityClayton, VIC, Australia

**Keywords:** palatogenesis, cleft palate, mouse models, human development, nasal septum, hard palate, soft palate, uvula

## Abstract

Clefting of the secondary palate is one of the most common human birth defects and results from failure of the palatal shelves to fuse during embryonic development. Palatogenesis is traditionally considered to be a highly conserved developmental process among mammalian species. However, cleft palate phenotypes in humans are considerably more variable than those seen in mice, the most common animal model for studying palatal development and pathogenesis of cleft palate. In this investigation, we utilized macroscopic observations, histology and 3D imaging techniques to directly compare palate morphology and the oral-nasal cavity during palate closure in mouse embryos and human conceptuses. We showed that mouse and human palates display distinct morphologies attributable to the structural differences of the oral-nasal cavity. We further showed that the palatal shelves interact differently with the primary palate and nasal septum in the hard palate region and with pharyngeal walls in the soft palate region during palate closure in mice and humans. Knowledge of these morphological differences is important for improved translation of findings in mouse models of human cleft lip/palate and, as such, should ultimately enhance our understanding of human palatal morphogenesis and the pathogenesis of cleft lip/palate in humans.

## Introduction

The palate forms the roof of the mouth and separates the oral and nasal cavities in humans. In mammals, the palate is formed from two distinct components: the primary and the secondary palates. The primary palate constitutes the most anterior part of the palate (anterior to the incisive foramen) and is formed through posterior expansion of the frontal-nasal process. The secondary palate constitutes the majority of the palate (posterior to the incisive foramen) and is formed through fusion of paired palatal shelves that derive from medial outgrowths of the maxillary processes. Development of the secondary palate is a multi-step process that includes palatal shelf growth, elevation and fusion (Greene and Pratt, 1976; Ferguson, 1988). Defects in any stage of palate development can result in cleft palate, one of the most common human birth defects. Cleft palate (CP) is commonly seen as a co-morbidity with cleft lip, but it may present as an isolated anomaly (Mossey et al., 2009; Rahimov et al., 2012). In addition, CP is seen as a clinical feature in excess of 400-named human syndromes (Murray and Schutte, 2004). Non-syndromic cleft palate, which affects one in every 1,500–2,000 live births worldwide, can have a complex etiology, being caused by genetic abnormalities that have either a primary or secondary affect on palatal development, environmental factors or the combination of both (Dixon et al., 2011; Burg et al., 2016). In recent years, significant progress has been made in identifying genetic contributors to cleft palate through human genetic studies (Cobourne, 2004; Stanier and Moore, 2004; Marazita, 2012; Leslie and Marazita, 2013).

As palatogenesis occurs in all mammalian species, our basic understanding of palatal morphogenesis comes principally from research conducted in various animal models. Human embryogenesis is commonly divided into 23 Carnegie Stages that cover the first 8 post-conceptal weeks of development (O'Rahilly and Müller, 2011). Palatogenesis in humans occurs relatively late during embryogenesis. In humans, the primordia of the secondary palate appear as outgrowths of the maxillary process at the end of the 6th week of embryonic development. Subsequent growth in the 7th week leads to formation of vertically oriented palatal shelves on both sides of the tongue. The palatal shelves elevate to the horizontal plane above the dorsum of the tongue in the 8th week (the end of embryogenesis) and fuse with each other to form an intact palate in the 9th week (the beginning of fetal development) (Burdi and Faist, 1967; Sperber, 2002). Compared to human palatogenesis that spans several weeks during development, mouse palatogenesis can be completed in several days. In mice, secondary palate development starts at embryonic day 11.5 (E11.5) followed by vertical growth to form the palatal shelves flanking the tongue. Palatal shelf elevation occurs in a narrow time window between E14- E14.5 and palatal shelf fusion is completed at E15.5 (Walker and Fraser, 1956; Gritli-Linde, 2007; Bush and Jiang, 2012). With advances in genetic technologies, mouse models have been widely used for studying the etiology and pathogenesis of cleft palate (Gritli-Linde, 2007; Bush and Jiang, 2012). Targeted mutagenesis in mice has shown that cleft palate can result from mutations in genes that encode proteins with a diverse array of functions, including signaling proteins and receptors, transcription factors and nuclear proteins, cytoplasmic and membrane-bound proteins and extracellular matrix components (Gritli-Linde, 2008). This diversity of molecular players highlights the complexity of cellular functions required for correct morphogenesis of the palatal shelves and the other facial structures that influence palatal development. Even despite the enormous benefits of research utilizing mouse models facilitating our understanding of the genetic complexities of palate morphogenesis (Gritli-Linde, 2008; Bush and Jiang, 2012), it remains unclear if many clefting genes in mice could cause cleft palate in humans. To some degree, this may be due to the typically more severe (homozygous) loss of function alleles that are often studied in mice. However, differences in the developing facial form, anatomical structure, and in the timing of palatogenesis itself may also be contributing factors. Past studies have suggested that palate development is not only regulated by factors intrinsic to the palatal shelves, such as gene-regulated cellular activities, but that it also relies on factors extrinsic to the palatal shelves such as tissue interactions between the palatal shelves, tongue, mandible and maxilla (Greene and Pratt, 1976; Ferguson, 1978). Considering the pronounced morphological differences between the mouse and human head and face, it is possible that some human cleft phenotypes result from failure of tissue interactions that are specific to human palatogenesis. In this study, we conducted a detailed morphological comparison between the mouse and human palate at the stages of palate closure. We identify multiple morphological factors that may be important for palate development in humans. These differences highlight the considerations that should be made when translating findings in models organisms such as the mouse to the pathogenesis of cleft palate in humans.

## Materials and methods

### Specimen collection

Mice were maintained on a C57BL/6J × 129X1/SvJ mixed genetic background. Female mice were sacrificed at 14.5, 15.5, 16.5, or E18.5 days post-coitum. The morning when a vaginal plug was observed was defined as embryonic day 0.5 (E0.5). Embryos were dissected from the uterus in PBS, fixed in 4% paraformaldehyde overnight at 4°C, and stored in 70% ethanol. For gross assessment of the palatal shelves, the tongue and lower jaw were removed from specimens. As reported previously (Yu and Ornitz, 2011), E14.5 specimens displayed the most variable palate morphology due to the rapid transitions of the elevation process occurring around this time. Specimens with horizontally orientated palatal shelves were selected for further morphological analysis. At least three specimens were examined at each stage. All mouse work was approved by the Animal Care and Use Committee of the Seattle Children's Research Institute.

Human conceptal tissues were recovered under an approved protocol through the University of Washington (IRB Approval No. STUDY00000380). The age for human specimens was estimated by a combination of gestational ultrasound and fetal foot length measurements where possible (Shepard, 1975; FitzSimmons et al., 1994). The gender was determined by either anatomic identification or PCR detection of Y-specific sequences using standard methods. As human palatogenesis spans from late embryogenesis to early fetal development, we selected specimens with the ages ranging from 50 to 70 days post conception (8–10 weeks of human embryonic development) in this study. By observing the gap between the opposing shelves, we divided human specimens into three age groups that show different progress on palate closure. Specimens at the ages of 53 days (*n* = 2) and 54 days (*n* = 4) showed an open palate with a large gap along the AP length. Specimens at the ages of 56 days (*n* = 2), 57 days (*n* = 7) and 58 days (*n* = 2) showed a partially fused palate with a small gap at the posterior end region. Specimens at 59 days of age showed various morphologies at the posterior end region that included a small posterior gap (*n* = 3), split uvula (*n* = 2) or complete fusion (*n* = 1). The palate was completely fused in specimens at the ages of 63 days (*n* = 1), 64 days (*n* = 1) and 67 days (*n* = 3). Human palate morphology at each age examined was largely consistent with that of a recent study except that we did not observe any substantial palatal shelf fusion at the age of 54 days (Danescu et al., 2015).

### Histology

Mouse and human craniofacial tissue was embedded in paraffin and sectioned either in the coronal or sagittal plane. 4 μm paraffin sections were stained with hematoxylin and eosin (H&E) for morphological analysis. Von Kossa staining was used to detect mineralized bone tissues as described previously (Yu et al., 2003).

### Optical tomography and micro-computed tomography

Optical projection tomography (OPT) and micro-computed tomography (microCT) were conducted in the Small ANimal Tomographic Analysis (SANTA) Facility at the Seattle Children's Research Institute. Samples for OPT were prepared and imaged under UV light as described in Zovein et al. (2010) using a Bioptonics 3001M scanner. MicroCT imaging was performed using a model 1,076 *in vivo* scanner (Skyscan, Belgium). Standard settings were used for imaging of craniofacial bones (Vora et al., 2016). For human soft tissue imaging using the microCT, tissues were first stained with PTAH (phosphotungstic acid-hematoxylin) as previously described (Siebert et al., 2013). Raw OPT and microCT scan data were reconstructed using NRecon V1.6.9.4 software (Skyscan, Belgium) and imported into the Drishti Volume Exploration software V 2.6.1 for 3D rendering and image capture.

## Results

### Palatal shelf shape and their position in the oral-nasal cavity

**Figure 1:**
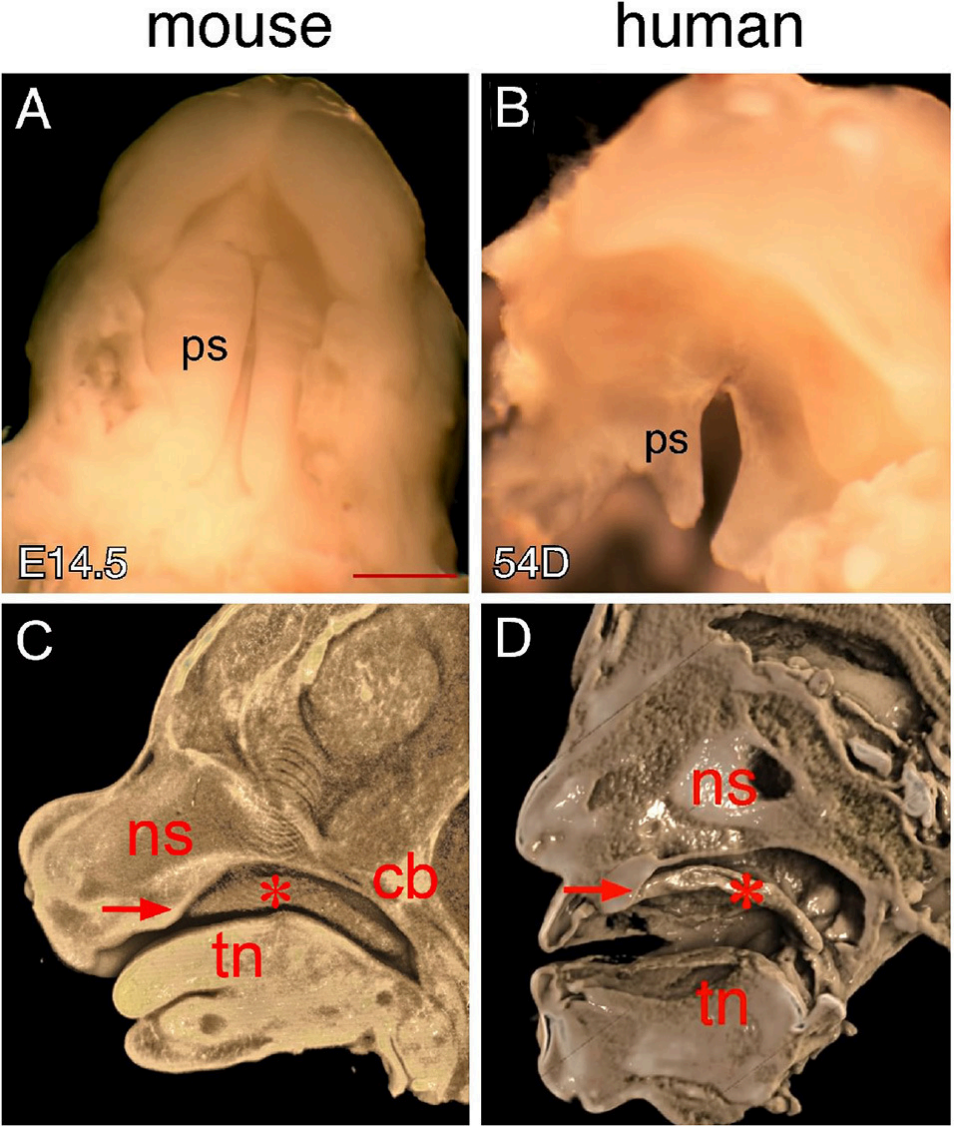
**Comparison of mouse and human secondary palates. (A)** Inferior view of newly elevated palatal shelves of an E14.5 mouse embryo. **(B)** Inferior view of newly elevated palatal shelves of a 54-day human embryo. **(C)** Virtual mid-sagittal cut through a 3D-rendered Optical Projection Tomography (OPT) scan of an E14.5 mouse embryonic head. **(D)** Virtual mid-sagittal cut through a 3D rendered microCT scan of a PTAH-stained 54-day human embryonic head. Asterisks in **(C,D)** indicate elevated palatal shelves. Arrows in **(C,D)** indicate the junction between the primary and secondary palate. cb, cranial base; ns, nasal septum; ps palatal shelf; tn, tongue. Scale bar in **(A)** for **(A,B)**, 1 mm.

Palatal shelf elevation marks the onset of palate closure, during which the palatal shelves overcome the tongue obstruction and move to a horizontal position for contact and fusion at the midline of the oral-nasal cavity. Despite differences in the conceptual age between mouse and human embryos at the time palatal shelf elevation occurs, both embryos are actually at a similar developmental stage during embryogenesis (Theiler Stage 22 in mice and Carnegie Stage 20–21 in humans) when most of the craniofacial structures including the primary palate, nasal cavity and tongue have been fully developed (Theiler, 1989; Kaufman, 1992). We therefore started morphological comparison using specimens with newly elevated palatal shelves. Examination of palate morphology in mice indicate that the newly elevated opposing shelves at E14.5 are not in contact with each other but are initially separated by a narrow gap along the anterior-posterior (AP) axis (Figure 1A), which is closed through continual shelf growth in the horizontal plane (Bush and Jiang, 2012). When examining human palates of different ages, we found that the palatal shelves of 54-day embryos have elevated but remain separated by a gap along the AP axis (Figure 1B). These observations indicate that the human palatal shelves do not contact each other immediately after elevation and that palatal shelf fusion in humans may also require continual shelf growth in the horizontal plane that occurs in mice.

To determine craniofacial tissue interactions during palate closure, we examined the position of the elevated shelves in an intact oral-nasal cavity environment using 3D imaging that allows multi-axis analysis of each specimen. Generally, the elevated shelves are located between the nasomaxillary complex and the tongue. However, mouse and human palatal shelves show distinct relationships with these craniofacial tissues in the oral-nasal cavity. For example, the elevated shelves are entirely inferior to the nasomaxillary complex in humans (Figure 1D), but are inferior to both the nasomaxillary complex and cranial base in mice (Figure 1C). While mouse palatal shelves are superior to the tongue, human palatal shelves dramatically change their orientation in the posterior region, toward the tongue base (Figures 1C,D). These morphological differences are likely secondary to the anatomic differences between mouse and humans. In mice, the nasomaxillary complex is more anteriorly positioned in the head, which brings the cranial base and brain into an adjacent position with the elevated shelves, tongue and mandible (Figure 1C). In humans, the nasomaxillary complex is more dorsally positioned and is aligned with the tongue and mandible along the dorsal-ventral (DV) axis, which causes the elevated shelves to end in a distinct pharyngeal space posterior to the tongue (Figure 1D).

### Palatal regions along the AP axis in mice

**Figure 2:**
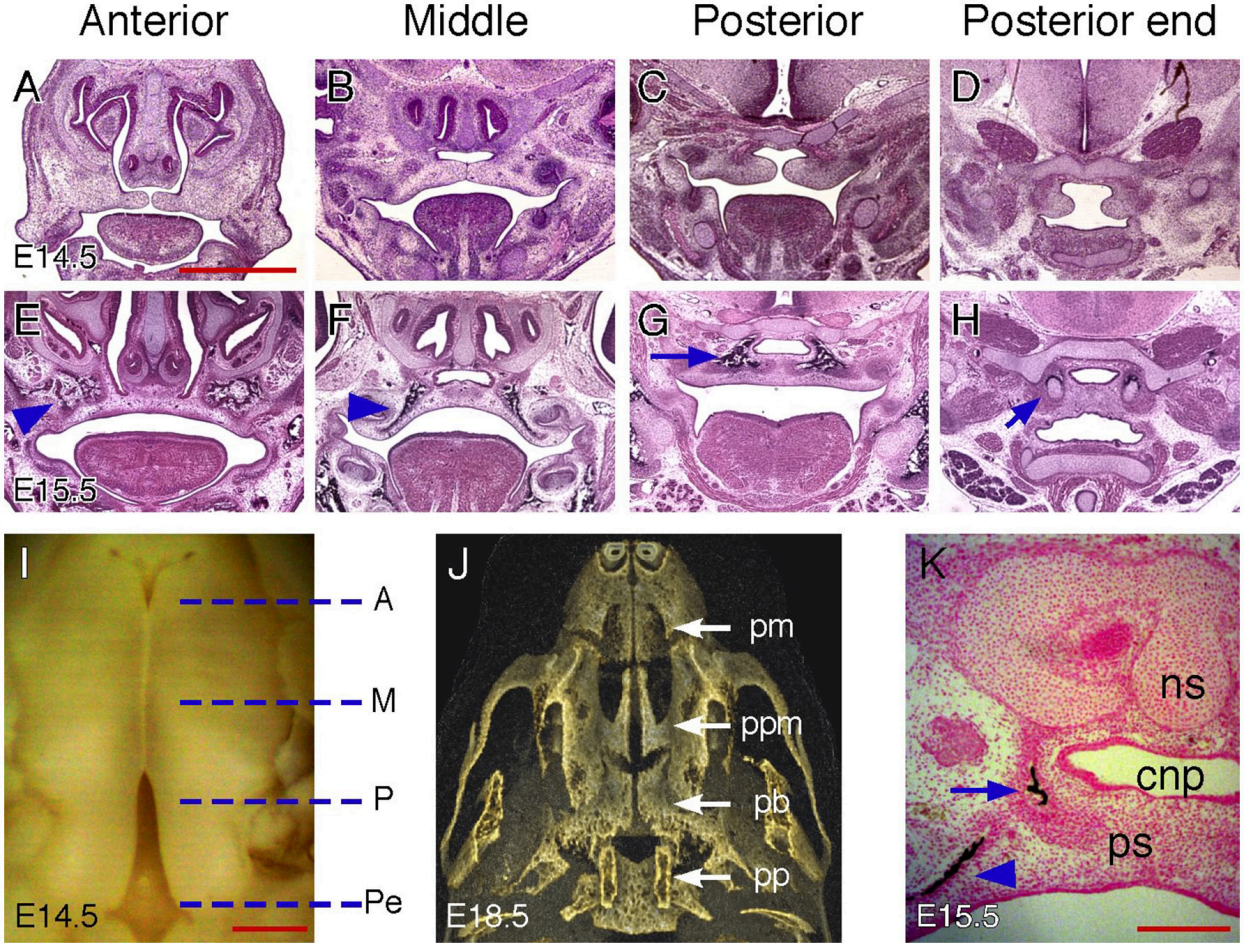
**Palatal regions along the AP axis in mice. (A–D)** H&E-stained coronal sections of an E14.5 mouse embryonic head showing anterior, middle, posterior, and posterior end regions. **(E–H)** H&E stained coronal sections of an E15.5 mouse embryonic head showing anterior, middle, posterior and posterior end regions. Arrowheads in **(E,F)** indicate maxillary ossification. Arrow in **(G)** indicates palatine bone ossification. Arrow in **(H)** indicate cartilages in the pterygoid process. **(I)** Inferior view of partially fused palatal shelves of an E14.5 mouse embryo. Dotted lines indicate the equivalent position of coronal sections shown in **(A–H)**. A, anterior; M, middle; P, posterior; Pe, posterior end. **(J)** Inferior view of a 3D rendered microCT scan of an E18.5 mouse embryonic head showing skeletal patterns in the palate. pm, premaxilla; ppm, palatine process of the maxilla; pb, palatine bone; pp, pterygoid process. **(K)** Von Kossa-stained coronal section of an E15.5 mouse embryonic head showing both maxillary (arrowhead) and palatine bone (arrow) ossification in the middle region. cnp, common nasal passage; ns, nasal septum; ps, palatal shelf. Scale bars in **(A)** for **(A–H)**, 1 mm; in **(I)**, 500 μm; in **(K)**, 200 μm.

Studies of mouse palatogenesis have suggested that different palatal regions along the AP axis display distinct morphological changes that may result from region-specific gene expression during development (Brinkley and Vickerman, 1979; Hilliard et al., 2005; Okano et al., 2006; Bush and Jiang, 2012). The mouse palate is often geometrically divided into three (anterior, middle and posterior) regions (Bulleit and Zimmerman, 1985) or four (anterior, middle, posterior and posterior end) regions along the AP axis (Figure 2I) (Brinkley and Vickerman, 1979, 1982; Okano et al., 2006; Yu and Ornitz, 2011). In a previous study, we examined shelf morphological changes during the course of palatal shelf elevation and described anatomic landmarks from coronal sections of E14.5 mouse palates for identifying each palatal region along the AP axis (Yu and Ornitz, 2011). We found that these landmarks are also applicable to the palate at a later stage (E15.5) when palatal shelf fusion is complete. One of the most distinct features of the anterior region is the presence of paired nasal meatuses flanking the nasal septum that open toward the oral cavity before elevation. After elevation, fused palatal shelves seal off this nasal opening, separating the nasal and oral cavities (Figures 2A,E). In the middle region, the nasal septum remains present on coronal sections but the nasal meatuses are not evident due to lateral fusion of the nasal septum with the maxillary process, which separates the nasal cavity from the oral cavity before elevation (Figures 2B,F). Palatal shelf fusion in this region creates an additional space, which has been previously been referred to as the common nasal passage (CNP) (Ferguson, 1978). A major change of anatomic landmarks in the posterior region is the appearance of the cartilaginous cranial base on coronal sections due to posterior extension of the palatal shelves beyond the nasomaxillary complex (Figures 1C, 2C,G). The CNP remains after palatal shelf fusion, extending all the way to the posterior end region. In the posterior end region, the palatal shelves extend into the space between the cartilaginous cranial base and hyoid bone (Figures 2D,H). The tongue, which is visible on coronal sections of the anterior, middle and posterior regions, is no longer present on coronal sections of the posterior end region.

The secondary palate can also be divided into the hard and soft palate along the AP axis due to differential ossification and myogenesis occurring in the palatal mesenchyme. The hard palate is formed by the palatine process of the maxilla and the horizontal plate of the palatine bone. The soft palate does not have bones but contains several muscles covered by the mucous membranes. In the fully developed mouse palate, the palatine process of the maxilla is located posterior to the premaxilla and inferior to the vomer of the nasal cavity (Figure 2J). The palatine bone is more posteriorly located and inferior to the presphenoid of the cranial base. The palatal region posterior to the palatine bone is the soft palate, which is inferior to the basisphenoid of the cranial base and flanked by paired pterygoid processes. However, ossification within the palatal mesenchyme is not evident at the time palatal shelf elevation occurs (E14.5). Therefore, it is not clear how the earlier geometrical palatal division correlates with the palatal mesenchymal differentiation that leads to formation of the hard and soft palate. We therefore examined coronal sections of mouse palates at E15.5 when intramembranous ossification becomes clearly visible in the palatal mesenchyme. Based on the observed ossification patterns, we suggest that the anterior and posterior regions correspond to the palatine process of the maxilla and palatine bone, respectively (Figures 2E,G). The palatine process of the maxilla remains present in the middle region (Figure 2F), but is reduced to a remnant that is co-present with the palatine bone on the coronal sections (Figure 2K). As the pterygoid bones are formed through endochondral ossification, their cartilaginous templates appear on coronal sections of the posterior end region (Figure 2H), suggesting that the region demarcated as the posterior end in earlier stages corresponds to the soft palate at later stages.

### Palatal regions along the AP axis in humans

**Figure 3:**
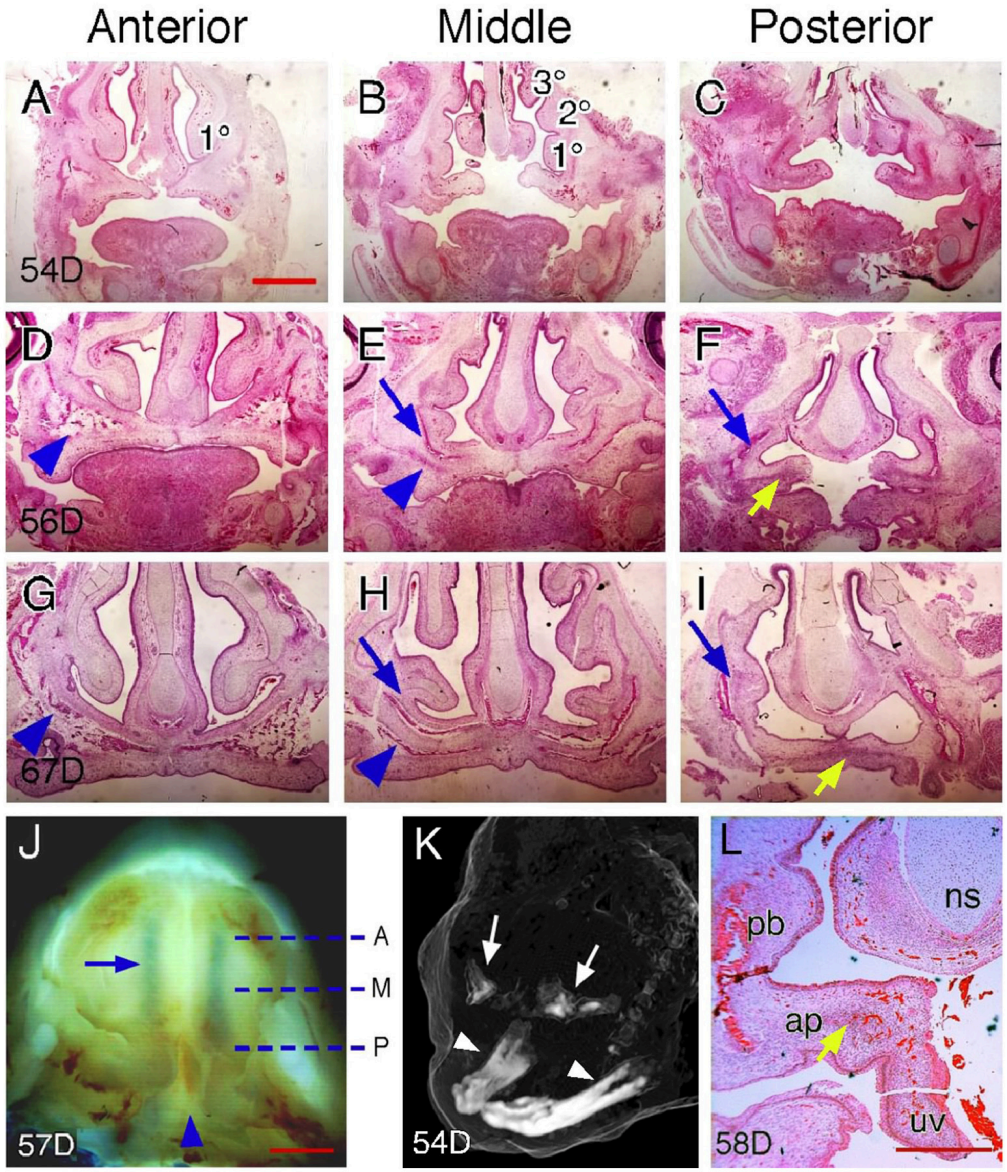
**Palatal regions along the AP axis in humans. (A–C)** H&E-stained coronal sections of a 54-day, **(D–F)** a 56-day, and **(G–I)** a 67-day human fetal head showing anterior, middle and posterior regions. Arrowheads in **(D,E,G,H)** indicate maxillary ossification. Blue arrows in **(E,F,H,I)** indicate palatine bone ossification. Yellow arrows in **(F,I)** indicate aponeurosis. 1°, inferior nasal concha; 2°, middle nasal concha; 3°, superior nasal concha. **(J)** Inferior view of partially fused palatal shelves of a 57-day human conceptus. Dotted lines indicate the equivalent position of coronal sections shown in **(A–I)**. A, anterior; M, middle; P, posterior. Arrowhead indicates the posterior end region. Arrow indicates the inferior nasal meatus. **(K)** Oblique view of a 3D-rendered microCT scan of a 54-day human embryonic head showing initial stages of ossification of the maxillae (arrows) compared to the advanced ossification of the mandible (arrowheads). Note: the soft tissue surface of the head is rendered as a “ghost” image for context. **(L)** H&E-stained coronal section of a 58-day human fetal head showing the posterior end region. Arrow indicates aponeurosis. ap, aponeurosis; ns, nasal septum; pb, palatine bone; uv, uvula. Scale bars in **(A)** for **(A–I)**, 1 mm; in **(J)**, 1 mm; in **(L)**, 500 μm.

It is not known if the human palate has regional heterogeneity demarcated by gene expression patterns similar to that seen in the developing mouse palate. In a recent study, anatomic landmarks were used to identify the junction between the hard and soft palate and to define the subregions of the soft palate (Danescu et al., 2015). We further examined serial coronal sections of human palates at different stages of the closure process to identify region-specific landmarks of the hard palate and compared these with similarly landmarked mouse palates. We found that paired nasal meatuses superior to the palate are much longer in humans than those of mice and expand over almost the entire palate during shelf closure. We therefore used the anterior end, middle and posterior end of the nasal meatus to define the anterior, middle and posterior region of the human palate, respectively (Figure 3J). The human palate also has a distinct posterior end region, which is characterized by a change in palatal curvature and formation of the uvula.

Examination of coronal sections of human palates indicates that anatomic landmarks used for identifying mouse palatal regions are not completely applicable in humans. For example, the nasal septum, which only appears in the anterior and middle regions of the mouse palate, is visible on coronal sections of every palatal region in humans (Figure 3). We found that the number of nasal conchae shown on coronal sections can be used to identify palatal regions in humans. In the anterior region, only the inferior nasal concha is visible on coronal sections (Figures 3A,D,G). The number of nasal conchae increases posteriorly and all three (superior, middle and inferior) conchae appear on coronal sections in the middle region (Figures 3B,E,H). The nasal conchae no longer appears on coronal sections of the posterior region, but fusion of the nasal septum with the maxillary process can be observed (Figures 3C,F,I). It should be noted that such a fusion occurs in the middle region of mouse palates but in the posterior region of human palates. We found that the posterior end region of human palates shows the most variable morphology on coronal sections due to palatal curvature changes that could impact tissue rigidity and thus affect the sectioning plane. In some cases, the palatal shelves in the posterior end region become perpendicular to those of the posterior region (Figure 3L), indicating that the sectioning plane has been changed from coronal to transverse for the palatal shelves in the posterior end region.

We further examined ossification patterns in human palates at different stages of palate closure. Like in mice, mesenchymal ossification is not manifest in newly elevated human palatal shelves (Figures 3A–C) although ossification has initiated in the more lateral maxillary regions (Figure 3K). Region-specific ossification patterns can be observed in human palates that are undergoing fusion (Figures 3D–F) and in completely fused human palates (Figures 3G–I). Based on these ossification patterns, we suggest that the anterior region corresponds to the palatine process of the maxilla (Figures 3D,G). The middle region is a transition zone within the hard palate as the remnants of both the palatine process of the maxilla and palatine bone are found on the coronal sections (Figures 3E,H). In the posterior region, the palatine bone remains present but the palatine aponeurosis, a tendon-like tissue that serves as both the insertion site and origin for muscles of the soft palate, also appears on coronal sections (Figures 3F,I,L). This suggests that the posterior region begins at the transition point between the hard and soft palate, which leaves the curved posterior end region as the soft palate in humans.

### Tissue interactions during closure of the hard palate

**Figure 4:**
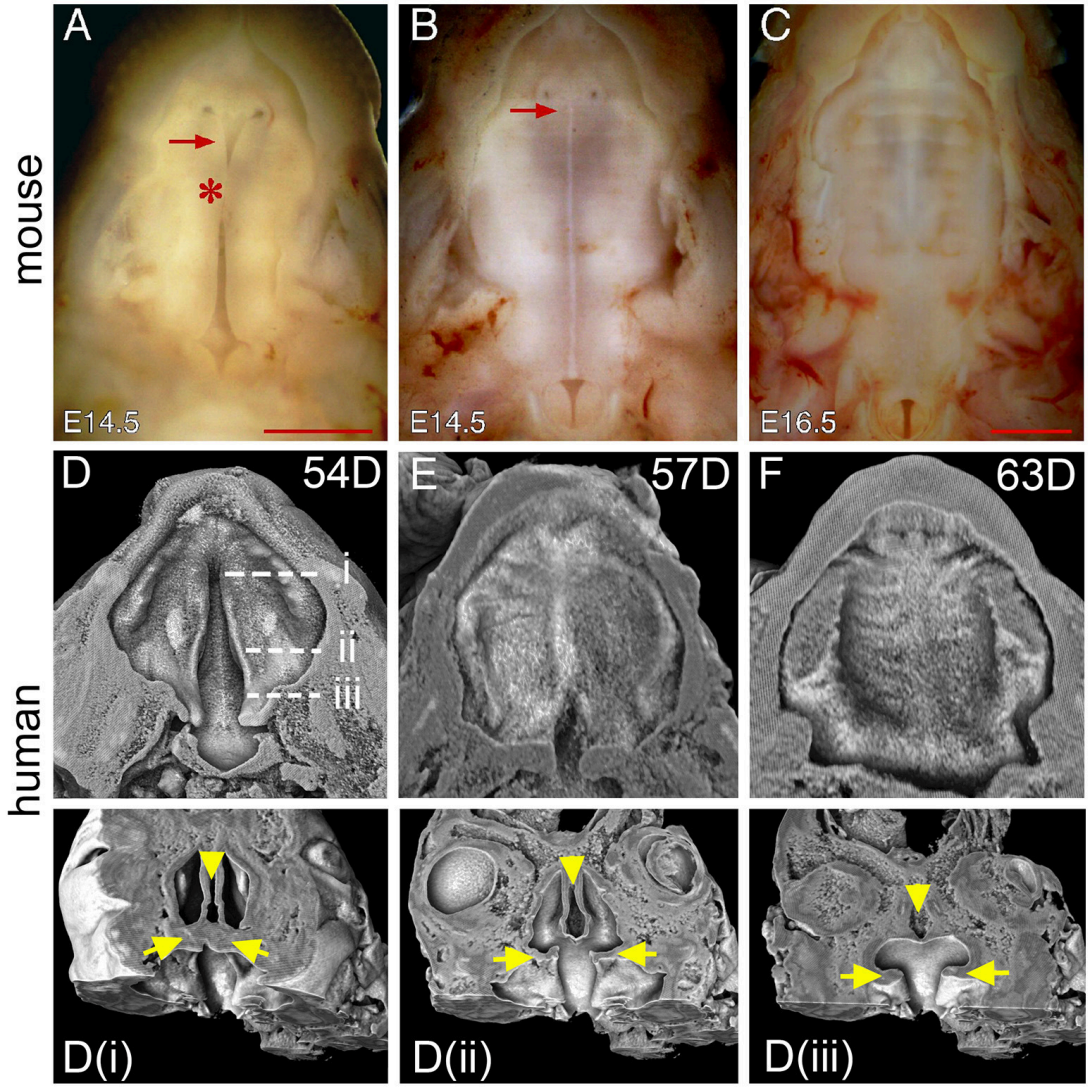
**Comparison of palate closure in mice and humans. (A)** Inferior view of the palatal shelves of an E14.5 mouse embryo showing initial contact at the middle region (asterisk). **(B)** Inferior view of a second E14.5 mouse embryo showing the formation of the MES after contact. Note that the MES appears as a white line in the middle. Arrows in **(A,B)** indicate a gap between the primary and secondary palate. **(C)** Inferior view of a completely fused palate of an E16.5 mouse embryo. **(D)** 3D rendered image showing unfused palatal shelf of PTAH-stained facial tissue from a 54-day human conceptus (the same one as in Figure 1D). Dotted lines indicate virtual segmentation planes along the AP axis to generate the coronal sections shown in **(Di–iii)**. Arrows indicate the palatal shelves and arrowheads indicate the nasal septum. **(E)** 3D rendered image showing partially fused palatal shelves of a PTAH-stained 57-day human conceptus. **(F)** 3D rendered image showing a completely fused palate of a PTAH-stained 63-day human conceptus. Scale bar in **(A)** for **(A,B)**, 1 mm; in **(C)**, 1 mm.

After elevation, continuous growth of the palatal shelves in the horizontal direction leads to the contact of opposing medial edge epithelium (MEE) and the formation of midline epithelial seam (MES), the degeneration of which results in continuity of the palatal mesenchyme and the completion of palate closure (Dudas et al., 2007; Iseki, 2011). In mice, the MEE contact first occurs at a position about a third of the way along the palate (the junction between anterior and middle region) (Figure 4A) and proceeds both anteriorly and posteriorly to form the MES along the entire AP length (Figure 4B). In mice, palatal shelf fusion is complete at E15.5 when the MES is completely degenerated (Figures 2E–H). During fusion, the mouse palate also shows a “Y”-shaped gap in the anterior end that reduces in size following MES formation and disappears at E16.5 (Figures 4B,C). The anterior gap, which is at the position of the incisive foramen, marks the boundary between the primary and secondary palate. Therefore, the fusion of the primary and secondary palate in mice occurs after palatal shelf fusion.

To examine human palate morphology during closure, we created 3D rendered images of human palates by removing the tongue and lower jaw through digital segmentation (Figures 4D–F), which can be further segmented along the AP axis to generate serial virtual coronal sections (Figures 4Di–iii). We examined human palates in three age groups, 53–54, 56–58, and 63–67 days, which represent the stages of before, during and after fusion, respectively. Compared to that of mice, the newly elevated human palate in specimens of the first age group shows a wider gap especially in the posterior region that appears as an inverted “V” shape (Figure 4D). The gap remains present in the posterior region as an intermediate morphology of palate closure in specimens of the second age group (Figure 4E). The posterior gap disappears from the human palate in specimens of the third age group (Figure 4F), which also shows degeneration of the MES in most of the palatal regions (Figures 3G–I). These observations suggest that palatal shelf fusion in humans proceeds from the anterior end to the posterior end that is slightly different from that of mice. Interestingly, we did not find the presence of the anterior gap in human palates of any of the ages examined. Examination of the palatal shelves in the sagittal plane indicates that the palatal shelves have already fused with the primary palate at the time of palatal shelf elevation in humans (Figure 1D). This does not occur in mice at the equivalent stage of palate development (Figure 1C).

**Figure 5:**
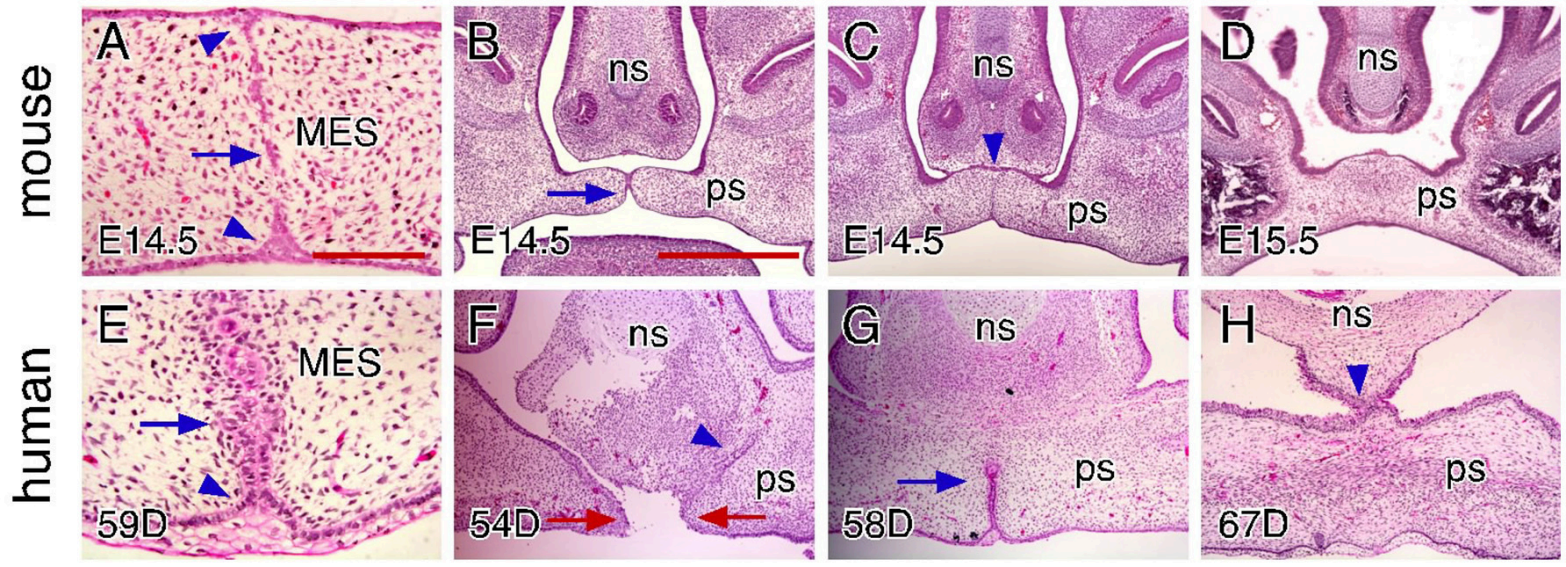
**Comparison of craniofacial tissue interactions during closure of the hard palate in mice and humans. (A)** H&E-stained coronal section showing MES degeneration in an E14.5 mouse palate. Arrow indicates epithelial islands and arrowheads indicate the epithelial triangles. **(B)** H&E-stained coronal section showing the contact of the palatal shelves in the anterior region (arrow) in an E14.5 mouse palate. Note that the palatal shelves are not in contact with the nasal septum. **(C)** H&E-stained coronal section showing epithelial seam formation between the palate and nasal septum in the anterior region (arrowhead) in an E14.5 mouse palate. Note the degenerated MES at the same region. **(D)** H&E-stained coronal section showing that the palate does not contact or fuse with the nasal septum in the posterior portions of the anterior region in an E15.5 mouse palate. **(E)** H&E-stained coronal section showing MES degeneration in a 59-day human palate. Arrow indicates epithelial rosettes and arrowhead indicates the alignment of the basal cells in the epithelial triangle. **(F)** H&E-stained coronal section showing epithelial seam formation between the palate and nasal septum in the anterior region (arrowhead) in a 54-day human palate. Note the gap between the opposing MEEs (red arrows). **(G)** H&E-stained coronal section showing partially (and dorsally) degenerated MES in the anterior region (arrow) in a 58-day human palate. Note that the palate completely fuses with the nasal septum. **(H)** H&E-stained coronal section showing that the palate is also in contact and fuses with the nasal septum in the posterior region in a 67-day human palate. Note the completely fused palate in the same region. ns, nasal septum; ps, palatal shelf. Scale bars in **(A)** for **(A,E)**, 100 μm; in **(B)** for **(B–D)**, and **(F–H)**, 500 μm.

It has been proposed that MES degeneration results from programmed cell death or epithelial-mesenchymal transformation of MEE cells that leads to MES breakdown to facilitate mesenchymal confluence between the opposing palatal shelves (Fitchett and Hay, 1989; Mori et al., 1994; Martinez-Alvarez et al., 2000; Cuervo and Covarrubias, 2004; Nawshad, 2008). Other studies in mice have suggested that MES degeneration involves collective radial MEE cell migration to form epithelial triangles on the dorsal (nasal) and ventral (oral) surfaces on the join, (Carette and Ferguson, 1992). In mice, the degenerating MES is also characterized by the presence of multiple epithelial islands and scattered mesenchymal bridging between the opposing palatal shelves in addition to the nasal and oral epithelial triangles (Figure 5A). We found that the MES in human palates shows cellular morphology distinct from that of mice. In the anterior and middle regions of human palates, the basal layers of the opposing MEE align to form the MES with only an oral epithelial triangle evident. As degradation is initiated, the MES converts into a string of multicellular epithelial rosettes (Figure 5E). These epithelial rosettes are gradually reduced in size and eventually dissipate in a dorsal-to-ventral order to complete MES degeneration and palatal shelf fusion (Figures 5E,G).

In addition to fusing with each other and the primary palate, the palatal shelves also fuse with the nasal septum. In mice, the fusion of the palatal shelves and nasal septum occurs only in a portion of the anterior region (Figures 5C,D), but not in the middle and posterior regions. We found that the palatal shelves are not in contact with the nasal septum at the time when the MES forms (Figure 5B). The contact of the palatal shelves and nasal septum also leads to the formation of an epithelial seam, which persists even after the MES has degenerated (Figure 5C). In humans, the fusion of the palatal shelves and nasal septum occurs at the time when the MEE of the newly elevated palatal shelves remains separated by a gap (Figure 5F). In the anterior and middle region, the epithelial seam between the palatal shelves and nasal septum degenerates more rapidly than the MES at the same region (Figure 5G), which is exactly opposite to that of mice (Figure 5C). In humans, the fusion of the palatal shelves and nasal septum extends into the posterior region, but as we found, may occur after palatal shelf fusion is complete (Figure 5H). By combining digital segmentation with 3D rendering, we assessed the position of the palatal shelves within the developing oral-nasal cavity of humans. We found that the MEE of the newly elevated palatal shelves is much closer to the nasal septum than to each other in the anterior and middle region (Figures 4Di,ii), but not in the posterior region (Figure 4Diii), which may explain the earlier contact and fusion of the palatal shelves and nasal septum in humans. The early fusion of the nasal septum with the shelves in humans may also dictate the distinctive dorsal to ventral dispersion of the palatal MES in humans. For example, the MES cannot migrate dorsally to integrate with the nasal surface as in mice as it has already disappeared following fusion with the nasal septum.

### Tissue interactions during closure of the soft palate

**Figure 6:**
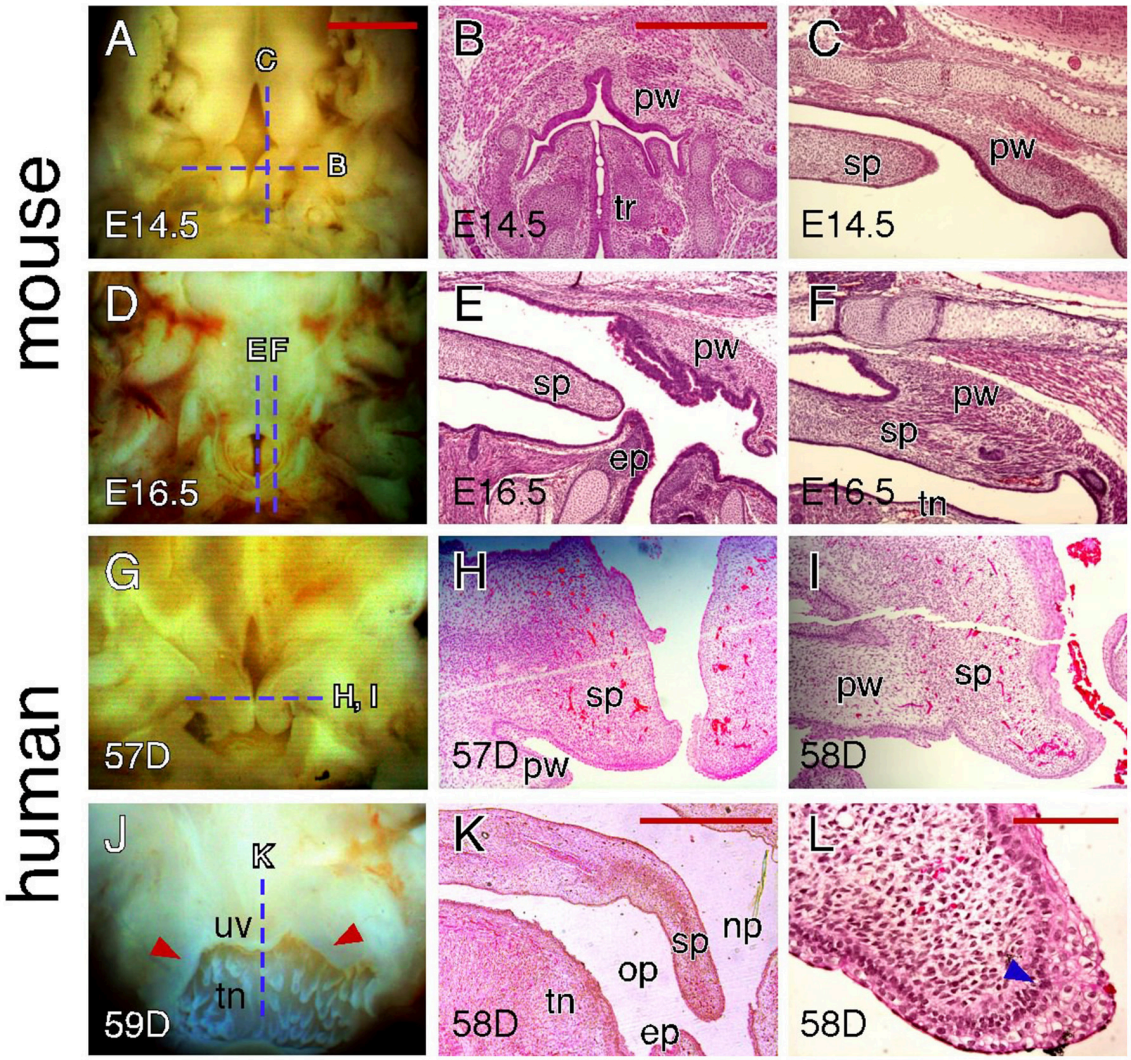
**Comparison of craniofacial tissue interactions during closure of the soft palate in mice and humans. (A)** Inferior view of the palatal shelves of an E14.5 mouse embryo showing the gap in the posterior end region. Dotted lines indicate the section planes represented in similarly aged (E14.5) embryos (shown in **B,C**). **(B)** H&E-stained coronal section showing outgrowths of the pharyngeal wall in an E14.5 mouse embryo. **(C)** H&E-stained para-sagittal section showing the posterior tip of the palatal shelves and pharyngeal outgrowth in an E14.5 mouse palate. **(D)** Inferior view of the palate of an E16.5 mouse embryo showing the opening between the pharyngeal walls. Dotted lines indicate the section planes represented in a similarly aged (E16.5) embryo (shown in **E,F**). **(E)** H&E-stained mid-sagittal section showing the interactions between the posterior tip of the palate and epiglottis in an E16.5 mouse palate. **(F)** H&E-stained para-sagittal section showing the fusion between the posterior tip of the palate and pharyngeal wall in an E16.5 mouse palate. **(G)** Inferior view of the palatal shelves of a 57-day human conceptus showing the gap at the posterior end region. Dotted line indicates the section plane represented in similarly aged (57 and 58-day) conceptuses (shown in **H,I**). **(H)** H&E-stained coronal section showing the unfused palatal shelves in the posterior end region of a 57-day human palate. **(I)** H&E-stained coronal section showing the mediolateral fusion of the palatal shelf and pharyngeal wall in the posterior end region of a 58-day human palate. **(J)** Posterior view of the fused palate of a 59-day human conceptus showing the uvula. Dotted line indicates the section plane represented in a similarly aged (58-day) conceptus (shown in **K**). Arrowheads indicate the palatopharyngeal arch. **(K)** H&E-stained mid-sagittal section showing that the posterior tip of a 58-day human palate is not in contact with the epiglottis. **(L)** H&E-stained coronal section showing the MEE at the posterior tip of a 58-day human palate. Arrowhead indicates the less-organized, multi-layered cuboidal epithelial cells. ep, epiglottis; op, oral pharynx; np, nasal pharynx; pw, pharyngeal wall; sp, soft palate; tn, tongue; tr, trachea; uv, uvula. Scale bars in **(A)** for **(A,D,G,J)**, 1 mm; in **(B)** for **(B,C,E,F,H,I)**, 500 μm; in **(K)**, 1 mm; in **(L)**, 100 μm.

In both mice and humans, a gap in the posterior end region can often be observed in certain aged specimens (E14.5 for mice and 56–58 days for humans). At gross observation, this gap looks similar in terms of the shape and size in both species (Figures 6A,G). However, further histological examination indicates that such a posterior gap is formed through different mechanisms in mice and humans. The gap formation in mice is not only due to the lack of fusion of the palatal shelves at the posterior end region but also due to the presence of paired outgrowths from the pharyngeal wall that are located posterior to the palatal shelves (Figures 6B,C). With the fusion of the palatal shelves, the posterior gap reduces in size but remains present (Figure 4B). This is because paired outgrowths of the pharyngeal wall do not fuse with each other and the remaining gap actually serves as the connection between the nasal passage and trachea (Figure 6B). At E16.5 when palatal shelf fusion is completed, the posterior tip of the palate is fused with pharyngeal outgrowths in the lateral region (Figures 6D,F) and is intimately positioned with the epiglottis in the middle region (Figure 6E). In contrast, the gap in humans is entirely due to the lack of fusion of the palatal shelves that show a distinct curvature at the posterior end region (Figures 6H,I). Completion of palatal shelf fusion results in the formation of the uvula as the posterior tip of the human soft palate (Figure 6J), which does not approximate with the epiglottis (Figure 6K). The palatal shelves at the posterior end region also fuse with the pharyngeal wall to become a part of the palatopharyngeal arch (Figures 6I,J). It should be noted that the fusion of the palatal shelves and pharyngeal wall occurs along the medial-lateral (ML) axis in humans and the uvula does not fuse with the pharyngeal wall. Interestingly, the MEE at the posterior tip of the human palatal shelves contains multiple layers of disorganized cuboidal epithelial cells (Figure 6L), which do not appear in the MEE at other regions of the human palate or in the MEE of the mouse palate.

## Discussion

In this study, we compared palate morphology between mice and humans at the time of palate closure. We find that mouse and human palates show distinct shelf morphological changes that at least in part could be due to differences in craniofacial structure between the species. Therefore, although palatogenesis is a conserved developmental process in mammals, the presence of species-specific factors can result in divergence in terms of the regulation of palate formation between species. As the mouse is the most commonly used animal model to study the mechanism of palate development and pathogenesis of cleft palate, our study has implications for the interpretation and translation of findings to humans.

### Identification of different palatal regions along the AP axis

Palate development is commonly studied on coronal sections of embryonic heads that allows researchers to visualize cellular and morphological changes within the palatal shelves and determine mediolateral differences on gene expression during palatal shelf growth, elevation and fusion (Bush and Jiang, 2012). The caveat of using coronal sections is regional heterogeneity in the developing palate, which requires tedious work to collect sections of different regions along the AP axis. Palate development especially at early stages is often studied in the anterior and posterior regions. However, coronal sections that represent the anterior and posterior regions can be very variable between different studies. In one extreme case, sections used for the anterior and posterior regions were indeed the posterior and posterior end regions described in our study, respectively (Wang et al., 2013). Our study of the anatomic landmarks provides a reliable way to select coronal sections of different regions along the AP axis. Further analysis of ossification patterns reveals developmental identities of these geometrically divided regions. For example the anterior and posterior regions correspond to the palatal process of the maxilla and palatine bones, two different portions of the hard palate, respectively, and the posterior end region corresponds to the soft palate. Therefore, similar palatal regions of mice and humans can be identified through species-specific anatomic landmarks for comparison of palate development in mice and humans.

### The role of craniofacial tissue interactions during pathogenesis of cleft palate

During palate closure, the palatal shelves not only contact with each other but also contact with the primary palate and nasal septum in the anterior region and contact with the pharyngeal wall in the posterior end region. Our study highlights species-specific craniofacial tissue interactions during palate closure in mice and humans, which have important implications for translating findings in mouse models.

Cleft palate is a common anomaly in both mice and humans. However, human cleft palate is typically viewed as encompassing a more variable presentation than that seen in mice. For example, human cleft palate phenotypes range from complete clefting to clefts of the soft palate or the uvula alone (Cobourne, 2004). However, partial cleft palate does not commonly occur in genetically modified mice and has been reported only in a few studies (Taya et al., 1999; Yu et al., 2005; Iwata et al., 2014). Although some part of this difference is likely due to less rigorous phenotypic analysis in mouse models, our finding that newly elevated human palate shelves are separated by a larger gap and have a more curved posterior part than that of mouse suggests that there could be a greater geometric obstacle for the palatal shelves to meet each other in the posterior part of human palate. This physical feature may increase the chance for cleft formation in the posterior part of the human palate.

Human cleft palate is often observed with cleft lip and cleft alveolus, which can occur either unilaterally or bilaterally. In fact, cleft lip and/or palate (CL/P) occurs more frequently than cleft palate only (CPO) in humans (Mossey, 2003; Genisca et al., 2009; Beaty et al., 2011; Dixon et al., 2011). On the other hand, a majority of genetically modified mice show CPO and those with CL/P are not only scarce but also almost always show other major craniofacial anomalies (Gritli-Linde, 2008). While differences in orofacial clefting between mouse models and humans have been extensively reviewed and discussed in the past (Gritli-Linde, 2008), our study of the interaction between the primary palate and secondary palate during palate closure provides additional insight into pathogenesis of orofacial clefting in humans. As the palatal shelves are fused with the primary palate prior to palatal shelf fusion in humans, in contrast to that in mice, primary palate development likely plays a greater role in secondary palate development in humans and may explain the markedly higher incidence of CL/P in humans.

Our observations also suggest that fusion of the palatal shelves and nasal septum plays a crucial role for human palate formation, but likely a lesser role in mouse. As fusion between the palatal shelves and nasal septum occurs prior to the fusion of the palatal shelves in humans, this may help to maintain the palatal shelves in the horizontal position that facilitates palatal shelf contact before fusion. Moreover, the extended fusion of the palatal shelves and nasal septum along the AP axis in humans could functionally separate the nasal and oral cavities even in the absence of palatal shelf fusion. This may explain the occurrence of unilateral cleft palate (in association with cleft lip) in humans as the palatal shelf on the unaffected side could be held in the horizontal position through the fusion with the nasal septum (Sperber, 2002).

### The role of craniofacial tissue interactions during soft palate development

The soft palate is the most posterior part of the secondary palate and consists of several muscles that are important for breathing, swallowing and speech (Grimaldi et al., 2015). A recent study of soft palate development in mice suggests that muscle insertion and orientation is similar to that in humans (Grimaldi et al., 2015). This is of particular interest given the apparent differences we have observed in the morphology of the mouse and human soft palate. For example, the uvula is formed at the posterior tip of the human soft palate but is absent in mice. Our study suggests that the distinct anatomical positioning of the palate and its interactions with pharyngeal wall contribute to the species-specific morphological differences in the soft palate. In mice, the soft palate remains dorsal to the tongue that forms a relatively straight pharynx. The posterior tip of the soft palate is either in contact with the epiglottis or fused with the pharyngeal wall, which prevents further posterior growth of the soft palate. In contrast, the human pharynx is more flexed due to the curvature of the tongue. The posterior tip of the human soft palate is neither in contact with the epiglottis nor fused with the pharyngeal wall and therefore forms the uvula.

While closure of the soft palate in mice is mediated by palatal shelf fusion (Walker and Fraser, 1956; Simley, 1975), it is less clear how the soft palate is closed in humans. It has been proposed that palate closure in humans involves two different morphogenetic mechanisms: epithelial fusion in the hard palate and epithelial merging in the soft palate (Burdi and Faist, 1967). A recent study of human soft palate morphogenesis suggests that palatal shelf fusion could also occur in the human soft palate, but MES removal in the human soft palate is much faster than that in the hard palate, which leads to the conclusion that human palatal shelf fusion in the hard and soft palates might be regulated by distinct AP signaling mechanisms (Danescu et al., 2015). We found that MES breakdown in the human hard palate involves the formation of distinctive multicellular epithelial rosettes and unidirectional movement of the MES to the oral palatal surface, both of which are not seen in mice. The notable rosette formation involves aligning the basal layers of the opposing MEE, which are usually covered by a layer of peridermal cells. On the other hand, the MEE at the posterior tip of the human soft palate show multiple layers of disorganized cuboidal epithelial cells, which suggests that the MEE interaction at the uvula could be different from that of other palatal regions in humans. Therefore, these rosette intermediates could be a key morphological marker for mechanistic studies of palate closure in humans.

## Author contributions

KY and TC conceived the study. All the mouse work was performed by KY. Coordination, collection and histological sectioning and staining of human conceptal tissues was performed by MD and TN. OPT and microCT imaging and analyses were conducted by TC. Coordination, oversight and management of the research was conducted by KY, TC, and IG. The manuscript was written by KY and TC.

### Conflict of interest statement

The authors declare that the research was conducted in the absence of any commercial or financial relationships that could be construed as a potential conflict of interest.
